# Simple and Low‐Cost Sampling of Cell‐Free Nucleic Acids from Blood Plasma for Rapid and Sensitive Detection of Circulating Tumor DNA

**DOI:** 10.1002/advs.201800614

**Published:** 2018-07-30

**Authors:** Choong Eun Jin, Bonhan Koo, Tae Yoon Lee, Kyudong Han, Seok Byung Lim, In Ja Park, Yong Shin

**Affiliations:** ^1^ Department of Convergence Medicine Asan Medical Center University of Ulsan College of Medicine 88 Olympicro‐43gil, Songpa‐gu ,05505 Seoul Republic of Korea; ^2^ Biomedical Engineering Research Center Asan Institute of Life Sciences Asan Medical Center 88 Olympicro‐43gil, Songpa‐gu ,05505 Seoul Republic of Korea; ^3^ Department of Technology Education and Department of Biomedical Engineering Chungnam National University 99 Daehak‐ro, Yuseong‐gu Daejeon 34134 Republic of Korea; ^4^ Department of Nanobiomedical Science & BK21 PLUS NBM Global Research Center for Regenerative Medicine Dankook University Cheonan 31116 Republic of Korea; ^5^ Department of Colon & Rectal Surgery Asan Medical Center University of Ulsan College of Medicine 88 Olympicro‐43gil, Songpa‐gu ,05505 Seoul Republic of Korea

**Keywords:** biooptical sensors, cell‐free nucleic acids, circulating tumor DNA, liquid biopsy, microfluidics platforms

## Abstract

Cell‐free nucleic acids (cfNAs) are emerging diagnostic biomarkers for monitoring the treatment and recurrence of cancers. In particular, the biological role and clinical usefulness of cfNAs obtained from the plasma of patients with various cancers are popular and still intensely explored, yet most studies are limited by technical problems during cfNA isolation. A dimethyl dithiobispropionimidate (DTBP)‐based microchannel platform that enables spontaneous cfNA capture in 15 min with minimal cellular background and no requirements for use of bulky instruments is reported first. This platform identified *KRAS* and *BRAF* hot‐spot mutations following cfDNA isolation from the blood plasma and tissues obtained from 30 colorectal cancer patients. The correlation of mutations between the primary tissues and plasma from the patients was high using this platform with whole genome sequencing compared to the spin‐column method. This platform can also be combined with various detection approaches (biooptical sensor, Sanger sequencing, and polymerase chain reaction (PCR)) for rapid, simple, low‐cost, and sensitive circulating tumor DNA detection in blood plasma. The efficiency and versatility of this platform in isolating cfNAs from liquid biopsies has applications in cancer treatment and precision medicine.

## 1 Introduction

Since the identification of cell‐free nucleic acids (cfNAs) in 1948, detection of cfNAs, including circulating tumor DNA (ctDNA), mRNA, and miRNAs, from liquid biopsies of human plasma has been employed for diagnosing various cancers, monitoring drug resistance, and quantifying minimal residual disease.^1^, ^2^, ^3^, ^4^ Liquid biopsies can overcome the limitations of tumor tissue biopsies, such as sampling bias, intratumoral heterogeneity, and difficulties in repeated extraction of samples.^5^, ^6^, ^7^ In particular, cfNAs are released in the blood by cell apoptosis and necrosis in both normal and cancer patients and can be considered as diagnostic markers.^2^ Among the cfNAs in blood plasma, ctDNA has been used for mutation genotyping in various cancers.^8^, ^9^ Because ctDNA exists at low levels in plasma, detection methods, such as the next‐generation sequencing (NGS), droplet digital polymerase chain reaction (PCR), and BEAMing, have been developed to analyze ctDNA with high sensitivity (0.01%–0.001%).^10^, ^11^, ^12^ However, these methods have not been considered for cfDNA sampling.^10^, ^11^, ^12^

One major technical issue in cfDNA analyses is the efficiency of the extraction procedure in obtaining the DNA from plasma. Most studies have used affinity column‐, magnet‐, and polymer‐based methods to perform cfDNA extraction.^1^, ^2^, ^3^ These methods are expensive, time‐consuming, and complex, and require additional instruments, such as a centrifuge or vacuum pump. In addition, they require chaotropic reagents for blood cell lysis that can lead to the release of DNA from noncancerous cells, which can strongly affect ctDNA analysis. Although recent cfDNA isolation methods that do not use centrifuges have been developed, they still need additional instruments, such as vacuum systems and DC power supplies for fluorescence labeling.^13^, ^14^, ^15^ To overcome this issue, procedures of cfDNA sampling from blood plasma need to be standardized to obtain a sufficient amount of DNA and reduce the cellular background, which would subsequently improve the detection sensitivity of ctDNA mutation profiling. However, these methods have been underexplored.

To the best of our knowledge, we developed a new strategy for simple and low‐cost sampling of cfNA for sensitive detection of ctDNA from the blood plasma of cancer patients. This method is based on the combination of dimethyl dithiobispropionimidate (DTBP) used as a nonchaotropic reagent, which contains an amine‐reactive homobifunctional imidoester (HI) and a central disulfide bond, and a microchannel platform to streamline the processing. We confirmed that DTBP directly binds to the amine group of nucleic acids by covalent bonding in the microfluidic platform without any dependence on a cell lysis step. Compared with the column‐based method, this DTBP platform rapidly isolates cfNA within 15 min and does not require bulky instruments (e.g., a centrifuge or a vacuum pump). We applied this DTBP platform to compare analyzed plasma with analyzed tumor tissue performed by *KRAS* and *BRAF* testing in 14 prospective colorectal cancer (CRC) patients (stages I–IV) and in 10 healthy controls. In addition, the DTBP platform was combined with a biooptical sensor, Sanger sequencing, and PCR‐based method, to obtain a low‐cost platform for ctDNA analysis that was validated in 11 retrospective CRC patients. This new platform offers a rapid, simple, low‐cost, and reproducible blood‐based profiling test.

## 2 Results and Discussion

### 2.1 Simple and Low‐Cost DTBP Platform for cfNA Sampling

**Figure 1:**
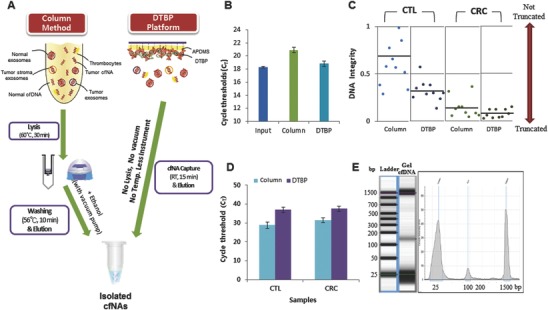
Simple and low‐cost cell‐free nucleic acid (cfNA) sampling for blood‐based testing. A) Schematic representation of the principle of cfNA isolation using the DTBP platform. Workflow of the column‐based method for cfNA isolation with a cell lysis step, high temperatures, and instruments (centrifuge and vacuum pump) (left). The DTBP platform can directly capture cfNA from plasma within 15 min without the requirements of a cell lysis step, high temperatures, or instruments (right). B) Comparison of the capture efficiency with the *Alu element* amplicon using the column‐based and DTBP platform. The error bars indicate standard deviations from the mean, based on at least three independent experiments. C) The integrity of isolated cfDNA using the column‐based method and the DTBP platform (CTL: 10 healthy control samples, CRC: 14 colorectal cancer samples). D) Real‐time PCR fluorescence signals for the amplified *Actin* gene (400 bp) with the isolated cfDNA using the column‐based method and the DTBP platform for checking the cellular DNA background. The error bars indicate the standard deviation from the mean, based on at least three independent experiments. E) Electrophoreogram of the isolated cfDNA using the DTBP platform. The lower peak is 25 bp and the upper peak is 1500 bp for size reference.

**Figure 2:**
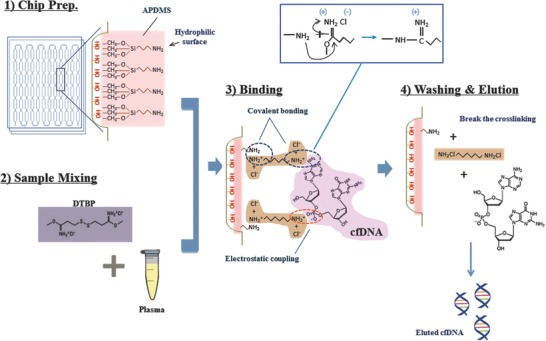
Operation principles of a cfNA isolation based microfluidic system with DTBP. 1) Chip preparation: assembling the microfluidic platform and inner surface modification with APDMS for binding the amine group of DTBP. 2) Sample mixing: blood plasma samples were mixed with DTBP solution (30 mg mL^−1^) and injected into the platform. 3) Binding: DTBP binds to the amine group of both APDMS and nucleic acids by covalent bonding and electrostatic coupling. 4) Washing and elution: after washing with PBS, elution buffer leads to the breakage of the cross‐linking, thus eluting cfDNA (or cfRNA).

**Figure 3:**
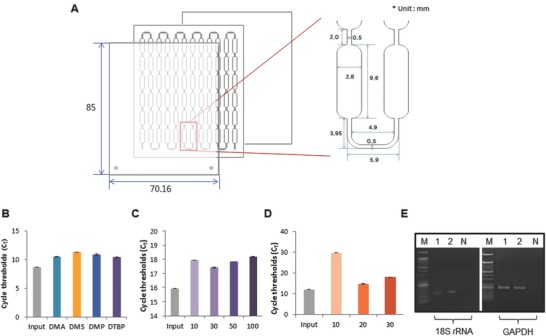
Characterization of the DTBP platform for simple and low‐cost cfNA isolation. A) The DTBP platform consists of several microwells in a single microfluidic chip. The width, depth, and length were optimized for cfNA isolation. B) The amplification efficiency of this platform is dependent on the type of homobifunctional imidoester reagents (dimethyl adipimidate (DMA), dimethyl suberimidate (DMS), dimethyl pimelimidate (DMP), and DTBP). The cfDNA capture rate is dependent on the C) DTBP concentration (mg mL^−1^) and D) APDMS concentration (µL mL^−1^). The error bars indicate the standard deviation from the mean, based on at least three independent experiments. E) cfRNA isolation from blood plasma using the DTBP platform. (M: size marker, 1: plasma #1, 2: plasma #2, N: negative control).

The cfNA (both cfDNA and cfRNA) isolation platform is based on the combination of a capture agent and a solid substance (**Figure** **1**). The cfNA isolation assay includes four steps: 1) chip surface modification, 2) sample mixing, 3) binding, and 4) washing and elution steps that can be performed in a single DTBP platform (**Figure** **2**). After the surface modification with 3‐aminopropyl diethoxymethylsilane (APDMS), the capture agent used is the nonchaotropic reagent DTBP for amine group‐mediated nucleic acid capture without any additional preparation (i.e., immobilization) prior to operation. DTBP has several methylene groups, disulfide linkage, and bifunctional imidoester groups.^16^ Similar to previous reports from our laboratory,^17^, ^18^ the chemical structure of DTBP is responsible for binding with the amine group of fragmented nucleic acids. The binding reaction between DTBP and cfNAs can be explained as follows: 1) the positively charged DTBP attracts negatively charged cfNA by electrostatic coupling, and 2) two imidoester groups in the structure of DTBP bind to the primer amine groups of nucleic acids to form amidine by covalent bonding (Figure 2). In order to collect the isolated cfNA, sodium bicarbonate (pH < 10.6) was then used as an elution buffer, since it can break the crosslinking of DTBP and cfNA complex from the surface of the platform (Figure 2). The solid substance used is a thin‐film microfluidic platform for the purification of cfNAs and DTBP complexes with a microchannel to streamline the processing (**Figure** **3**A). Use of the DTBP platform without a cell lysis buffer and instruments (Figure 1A) allows the isolation of cfNA from blood plasma within 15 min by overcoming the limitations of the column‐based method, such as the increased cellular background owing to cell lysis, the requirements of chaotropic reagents, large sample volume, and the use of instruments (i.e., vacuum pump and centrifuge).

We examined the capture efficiency of the column‐based method and the DTBP platform using the amplicon of the *Alu element* (115 base pair, bp), which was used to determine the integrity of cfDNA in blood (Figure 3B–D). We first compared the capture efficiency among the various HI reagents, such as DTBP and dimethyl adipimidate, dimethyl suberimidate, and dimethyl pimelimidate. The Ct value from the DTBP‐based platform was lower than that using other HI reagents (Figure 3B). The Ct value obtained using the DTBP method was also lower than that from the column‐based method, and was similar to the input as an absolute value (Figure 1B). Thus, we selected DTBP as an optimal capture agent and evaluated the optimal protocol for the isolation of cfDNA under the best experimental conditions (i.e., 30 mg mL^−1^ of DTBP and 20 µL L^−1^ of APDMS; Figure S1C,D, Supporting Information). We have also observed that cfRNA can be isolated using the conditions of the DTBP platform (Figure 3E).

The column‐based method uses a chaotropic reagent‐based lysis buffer, which leads to an increased cellular background and degrades the cfDNA. This consequently reduces the ctDNA detection rate.^3^, ^19^ To address this issue, we evaluated the utility of the DTBP platform for cfDNA isolation without the use of a lysis buffer and large instruments from 24 blood plasma samples, including 14 samples from prospective CRC patients, and 10 from healthy controls. The cfDNA concentration obtained from the DTBP platform was much higher than that obtained from the column‐based method (Table S2, Supporting Information). We examined the integrity of cfDNA and the amount of background cellular DNA from both the DTBP and column‐based methods. To measure the cfDNA integrity inplasma, we used two sets of *Alu element* primers, which amplified 115 and 247 bp products. A*lu* 247 bp represents the absolute amount of longer fragments of plasma DNA, whereas *Alu* 115 bp represents the total amount of cfDNA in plasma.^2^, ^20^ The DNA integrity was calculated using the *Alu* 247/115 ratio, with a ratio value that was close to 0, thus indicating that most of the DNA was truncated.^2^, ^20^ In Figure 1C, the *Alu* 247/115 ratio of cfDNA obtained from the CRC patients was lower than that obtained from healthy controls, and the cfDNA obtained from the CRC patients was more truncated with the DTBP platform than with the column‐based method. This result indicates that more cfDNA can be isolated from the DTBP platform (Figure 1C; Table S2, Supporting Information). Moreover, we amplified 420 bp of the *β‐actin* gene using qPCR to investigate the amount of background cellular DNA in the isolated cfDNA population. In the case of both healthy controls and CRC patients, the Ct value of the isolated cfDNA from the DTBP platform was more delayed than that from the column‐based method (Figure 1D; Table S2, Supporting Information). This means that the cellular background was smaller in value when the DTBP platform was compared to the case when the column‐based method was used. To evaluate the quality of isolated cfDNA from plasma using the DTBP platform, we analyzed the size of DNA fragments using the electrophoreogram in Figure 1E. The observed size of the cfDNA fragments was ≈165 bp (mean = 163 bp; range = 153–186 bp). Taken together, the simple and low‐cost sampling platform could isolate high‐quality cfNA at increased quantities from liquid biopsies of cancer patients.

### 2.2 Correlation of ctDNA Detection in Tissue and Liquid Biopsies of CRC Patients

**Figure 4:**
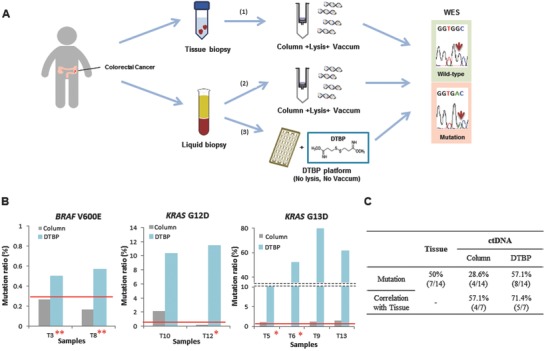
Application of the DTBP platform for cfDNA isolation and ctDNA analysis. A) Workflow scheme of 14 clinical samples, including primary tissues and blood plasma from colorectal cancer patients. 1) Mutation screening using the column‐based method for extraction from tissue biopsies and whole exome sequencing (WES) for detection. 2) Mutation screening using the column‐based method for extraction from blood plasma and WES for detection. 3) Mutation screening using the DTBP platform for extraction from blood plasma and WES for detection. B) Mutation ratio of the isolated ctDNA using the column‐based method (gray) and the DTBP platform (sky blue) for detecting *BRAF* V600E (left), *KRAS* G12D (middle), and *KRAS* G13D (right) mutations. The red line represents the cut‐off (criterion) for reporting a sample once the mutation (positive/negative) was detected. Two asterisks (*) represent samples from which the mutation was detected in only cfDNA and not in tissue DNA. Dual asterisks (**) represent samples from which mutations were detected only in cfDNA using the DTBP platform but not using the column‐based method. C) Correlation between WES results of primary tissues and plasma among 14 clinical samples with the ctDNA isolated using the column‐based method and the DTBP platform.

The clear advantage of the DTBP platform is that it can effectively and rapidly capture cfDNA. We further evaluated whether ctDNA could be sensitively detected from the cfDNA population. For this purpose, we prospectively collected the matched cancer tissues and blood plasma samples from14 CRC patients and compared the correlation of the mutation profiling between the column‐based method and the DTBP platform (**Figure** **4**A). We extracted genomic DNA from the tissues and identified cancer‐related mutations using whole exome sequencing (WES) methods. The WES results of the 14 studied samples are shown in Figure S1 of the Supporting Information. Ten of the 14 samples revealed various mutations, including known CRC‐related mutations, *BRAF* (two samples), *CTNNB1* (one sample), *KRAS* (five samples), *PIK3CA* (five samples), and *TP53* (one sample) (**Table** **1**). Subsequently, we used 500 µL of blood plasma samples from CRC patients to obtain cfDNA using both the DTBP and column‐based methods. To check the correlation of hot‐spot mutations (i.e., *BRAF* and *KRAS)* between the tissue and blood samples, *KRAS* mutations (G12D, G12V, and G13D), and a *BRAF* mutation (V600E) with a sequence‐specific synchronous coefficient of drag alteration (SCODA) mutation panel,^16^ were used in both methods (Table 1). In particular, the *BRAF* mutation identified in matched tissue samples was detected in two plasma samples (T3 and T8) using the DTBP platform but not using the column‐based method. The *BRAF* mutation that was investigated using the column method was under the detection limit of the SCODA calculation (Figure 4B, left) that was used tocompare quality and mutation ratios with WES results.^21^ In addition, the *KRAS* G12D mutation was detected in one sample (T10) by the column‐based method and in two samples (T10 and T12) by the DTBP platform. The *KRAS* G12D mutation in the T10 sample was identified using both methods, but the mutation ratio revealed by the DTBP platform was 4–10 times higher than that revealed by the column‐based method (Figure 4B, middle). Even the mutation in the tissue sample of T12 that could not be detected by the column‐based method was successfully detected by the DTBP platform that elicited an increased ratio (Figure 4B, middle). The *KRAS* G13D mutation was detected in three samples (T5, T9, and T13) by the column‐based method and in four samples (T5, T6, T9, and T13) by the DTBP platform. The mutation results of the two samples (T9 and T13) were correlated in tissues and plasma samples by both methods. Although the mutation could not be detected in the tissues of the two samples (T5 and T6), it was detected in either the column‐based method or the DTBP platform. The *KRAS* G13D mutation ratio was shown to be much higher by the DTBP platform than by the column‐based method in all four samples (Figure 4B, right). In several studies, plasma analyses have revealed *KRAS* mutations that were not seen in the tissues owing to sampling heterogeneities.^21^, ^22^ No mutations were detected in the 10 healthy plasma samples. We found a higher concordance in the statuses of *BRAF* and *KRAS* between primary tumor and plasma samples by the DTBP platform (71.4%) than by the column‐based method (57.1%) (Figure 4C and Table 1).

**Table 1:** Clinicopathological characteristics and sequencing results from primary tissue and plasma (ctDNA) samples obtained from 14 colorectal cancer patients. Bold text indicates detected mutations. WT: wild type

Nr.	Age	Gender	Pathologic Stage	Tissue [WES]	ctDNA	
					Column	DTBP
T1	68	M	2	WT	WT	WT
T2	62	M	2	WT	WT	WT
**T3**	54	M	4	***BRAF* (V600E)**	WT	***BRAF* (V600E)**
**T4**	62	M	3	***KRAS* (G12V)**	WT	WT
**T5**	42	M	4	WT	***KRAS* (G13D)**	***KRAS* (G13D)**
**T6**	42	M	2	WT	WT	***KRAS* (G13D)**
**T7**	48	F	4	***KRAS* (G12V)**	WT	WT
**T8**	34	F	4	**BRAF (V600E)**	WT	***BRAF* (V600E)**
**T9**	40	M	3	***KRAS* (G13D)**	***KRAS* (G13D)**	***KRAS* (G13D)**
**T10**	65	M	4	***KRAS* (G12D)**	***KRAS* (G12D)**	***KRAS* (G12D)**
T11	64	F	2	WT	WT	WT
**T12**	42	M	3	WT	WT	***KRAS* (G12D)**
**T13**	72	F	3	***KRAS* (G13D)**	***KRAS* (G13D)**	***KRAS* (G13D)**
T14	56	M	4	WT	WT	WT

### 2.3 Validation of Simple and Low‐Cost ctDNA Detection Platform

**Figure 5:**
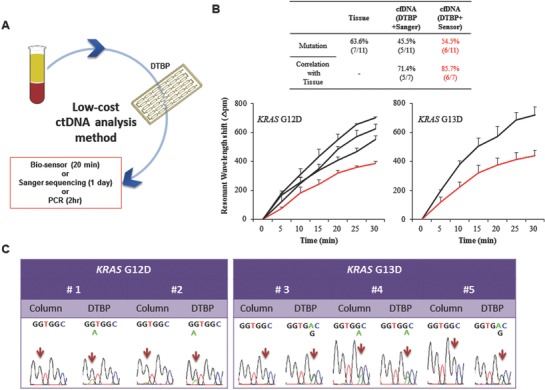
Simple and low‐cost ctDNA analysis for clinical diagnosis. A) Combination of the DTBP platform and Sanger sequencing for low‐cost ctDNA analysis. B) ctDNA was isolated from 11 plasma samples of colorectal cancer patients using the DTBP platform, and followed by the use of the biosensor for ctDNA analysis. The correlation between the primary tissues (OncoPanel result) and blood plasma (with the Sanger sequencing and the biooptical sensor) (up) was analyzed. Resonant wavelength shift using the biooptical sensor with either the G12D (down‐left) or the G13D mutant primers (down‐right). The error bars indicate the standard deviation of the mean, based on at least three independent experiments. C) Validation of this simple and low‐cost ctDNA analysis using five plasma samples in which mutations were not identified in the column‐based method.

Highly sensitive detection technologies, including NGS, are needed to detect ctDNA after cfDNA isolation because ctDNA exists at low levels in blood plasma. However, NGS is an expensive method and requires large plasma volumes (>10 mL) for ctDNA detection. To address this issue, we developed a simple and low‐cost ctDNA detection platform that combined the DTBP platform with Sanger sequencing, biooptical sensor, or PCR (**Figure** **5**A). The Sanger sequencing results of isolated cfDNA are shown in Figure S2A of the Supporting Information. When the Sanger sequencing was performed on one blood sample obtained from a CRC patient, the *KRAS* mutation G12D could be identified in the cfDNA that was isolated using only the DTBP platform (Figure S2B, Supporting Information). To validate the reproducibility of the DTBP platform, we repeated the process with plasma samples obtained from the same patients in different sample tubes and showed that the reproducibility of this platform was sufficient for use of the platform in clinical practice (Figure S2C, Supporting Information). Next, we tested 11 plasma samples (frozen and long‐term storage) of the CRC patients whose tissue samples showed hot‐spot mutations, as confirmed by the OncoPanel assay.^23^ The OncoPanel results of the tissue samples revealed a *BRAF* V600E mutation in one sample, *KRAS* G12D mutation in three samples, *KRAS* G12V mutations in two samples, and a *KRAS* G13D mutation in one sample. The cfDNAs obtained from 11 samples were isolated by the DTBP platform, and mutations were then analyzed using the Sanger sequencing or the biooptical sensor (Figure 5B). Using the simple and low‐cost ctDNA detection platform with Sanger sequencing, a *BRAF* V600E mutation was detected in one sample, a *KRAS* G12D mutation in two samples, a *KRAS* G12V mutation in one sample, and *KRAS* G13D mutations in two samples. This simple and low‐cost method with Sanger sequencing yielded a 71.4% correlation in the mutation profiles of the tissues and frozen blood plasma samples obtained from 11 CRC patients (Figure 5B; Table S3, Supporting Information). In addition, using the simple and low‐cost ctDNA detection platform with the biooptical sensor, which has been reported in cancer tissues samples,^24^ one *BRAF* V600E mutation, three *KRAS* G12D mutations, one *KRAS* G12V mutation, and one *KRAS* G13D mutation were detected in the plasma samples (Figure 5B). The correlation of mutation profiling between the tissues and frozen blood plasma samples from 11 CRC patients using the platform with the biooptical sensor was higher (85.7%) than that of the Sanger sequencing method (Figure 5B; Table S3, Supporting Information).

Finally, to validate the clinical utility of this simple and low‐cost platform, we tested five blood plasma samples prospectively collected from CRC patients. We identified *KRAS* G12D or G13D mutations using both the Sanger sequencing (Figure 5C) and PCR (Figure S2C, Supporting Information), but we could not identify them with the column‐based method. Although we could not confirm the mutations in the matched tissue samples because tissue samples were not available, *KRAS* G12D and G13D mutations were detected in all five plasma samples by the DTBP platform. These results showed that compared with the commercial column methods, simple and low‐cost sampling from the plasma of cancer patients via the DTBP platform is useful for the rapid detection of ctDNA mutations with high sensitivity.

## 3 Conclusions

Owing to the increasing usefulness of liquid biopsies in cancer applications, the cfNA analysis system, including the sampling and detection techniques, has been recently introduced. With the development of detection technologies, the development of a new cfNA‐sampling technique to overcome the high cost, slowness, low sensitivity, and complexity of the current methods was strongly desired. To address these issues, we developed a DTBP platform for simple and low‐cost cfNA isolation. This procedure only took 15 min without the use of chaotropic reagents, which lead to cfNA damage and increased cellular DNA background. In addition, the DTBP platform did not require bulky instruments for isolation.

Compared to the column‐based method, mutation profiling in tissues and plasma samples obtained from CRC patients was highly concordant with the use of the DTBP platform. In some cases, *KRAS* mutations were detected in plasma but not in the matched tissue samples owing to the increased ability of the DTBP platform to capture cfDNA. The mutations were not detected in the samples obtained from the healthy controls. In addition, cfDNA could be isolated with a low cellular background using the DTBP platform, and could be combined with traditional detection approaches, such as biooptical sensor (20 min), Sanger sequencing (1 d), and PCR (2 h) techniques, which are insufficient for ctDNA detection in plasma. Especially, the DTBP platform was combined with a biooptical sensor that can detect the ctDNA within 1 h from the time of extraction of blood plasma samples. The hot‐spot mutations were detected in the plasma by the DTBP platform, with >71% correlation between the tissues and frozen plasma samples obtained from the CRC patients.

Despite the advantages of this proof‐of‐concept platform, clinical trials with additional blood samples from patients with various cancers (e.g., colorectal, breast, and lung) would be needed to further establish the clinical utility of cfNAs. Undoubtedly, liquid biopsies will be used as diagnostic tools for human cancers. To achieve this, technical advances, including sampling and detection methods, should be standardized for introduction into large‐scale clinical trials. In addition, enhanced understanding regarding the use of cfNA as a biomarker in the diagnosis and treatment of cancers would be needed. Nevertheless, by combining it with cutting‐edge detection techniques, this simple and low‐cost sampling of cfNA via the DTBP platform could be useful for the clinical diagnosis and monitoring of cancer treatment.

## 4 Experimental Section

*Fabrication of cfNA Isolation Platform*: The simple and low‐cost cfNA sampling platform using DTBP comprises two 100 µm thin films (Kemafoil hydrophilic film, HNW‐100, COVEME, Italy) as outer layers, and a 300 µm thick double‐sided tape (Adhesive 300LSE‐9495LE, 3M, Minnesota, US) as an inner layer with 72 microwells in a single microfluidic chip (Figure S3A, Supporting Information). This platform (85 mm × 66 mm × 0.5 mm) was designed using AutoCAD (Autodesk, Inc., San Rafael, CA) and fabricated using a CO_2_ based laser‐cutting machine (VLS3.50‐Universal Laser Systems, Scottsdale, AZ).^17^, ^18^ The three‐layered microfluidic platform assembly was combined with DTBP for cfNA isolation from the blood plasma samples of 30 CRC patients and 10 healthy controls. To use DTBP as a cfNA capture probe with the microfluidic platform, the surface of the inner part of the microfluidic channel was treated with oxygen plasma (Covance Model, Femtoscience, Korea) for 10 min and then immersed in a solution of 2% solution of APDMS (Sigma‐Aldrich, St. Louis, MO) for 60 min at 65 °C to increase the hydrophilicity of the surface properties of the inner surface, followed by a thorough rinse with deionized water. The platform was then stored at room temperature until use.

*Operation of cfNA Isolation Platform*: The operation of the platform can be performed with the execution of four steps in a single chip: 1) chip preparation, 2) sample mixing, 3) binding, and 4) washing and elution (Figure 2). First, various concentrations of APDMS (1%, 2%, and 3%) and DTBP (10, 30, 50, and 100 mg mL^−1^) were examined in the reaction solution to set up the optimized condition for the interaction between the amine group of cfNAs and the amine group of the inner surface. 1) After the chip preparation with APDMS, 2) the plasma sample was briefly mixed with DTBP. 3) The mixture was then added into the chip. The cfNAs can bind with DTBP via covalent bonding and electrostatic coupling on the surface. 4)Finally, following the PBS washes executed to remove the debris from the plasma samples, the elution buffer was used to collect the cfNAs that were isolated within 15 min (Figure 2).

*Optimized Conditions of cfNA Isolation Platform*: To test and optimize the experimental conditions, 200 µL of the *ALU* gene (247 bp amplicon) was mixed with 100 µL of DTBP (100 mg mL^−1^) and then was injected into the platform. It was then placed at room temperature for 15 min to capture cfNAs from the samples. DTBP could directly capture the nucleic acids through a complex on the surface without the requirement of cell lysis buffer and bulky instruments. To compare the direct binding capability of cfNA, other HI groups were tested (100 mg mL^−1^), such as dimethyl adipimidate, dimethyl suberimidate, and dimethyl pimelimidate. All His were purchased from Sigma‐Aldrich (St. Louis, MO). After washing with phosphate‐buffered saline to remove debris from the samples using a syringe pump (100 µL min^−1^), an elution buffer (10 × 10^−3^ m sodium bicarbonate, pH < 10.6, flow rate 50 µL min^−1^) was used to collect the cfNAs that were isolated within a few minutes by breaking the crosslinking with DTBP and amine group of APDMS. The quantity and purity of the isolated cfNA were evaluated using the ratio of the optical densities of the samples with a Qubit fluorometer, a high sensitivity D1000 ScreenTape System (Agilent, Germany), and the dsDNA HS Assay Kit (Life Technologies, CA, USA). The DTBP platform was compared with the column‐based cfDNA isolation method (QIAamp Circulating Nucleic Acid Kit, Qiagen, Germany), which was used according to the manufacturer's protocol. To identify whether cfRNA can be isolated using the DTBP platform, two blood plasma samples were mixed with DTBP and DNase (Qiagen). The samples were injected into the platform, the debris was then washed away, and cfRNA was isolated. The *18S rRNA* and *GAPDH* genes were amplified with the isolated cfRNA and used to check the quality and quantity of cfRNA. The sequences of the primer set used in this study are described in Table S1 of the Supporting Information.

*Measurement of cfDNA Integrity and Cellular DNA Background*: To measure the cfDNA integrity, cfDNA was amplified with two types of *Alu* primer sets using real‐time PCR.^15^ The cfDNA samples were analyzed using the *Alu* 247/115 ratio. A ratio close to 1.0 indicated that the cfDNAs were not truncated, whereas that close to 0 indicated that cfDNAs were truncated. For real‐time PCR, the following procedure that was modified from the AriaMx real‐time PCR instrument protocol (Agilent Technologies) was used. 5 µL of cfDNA were amplified in a total volume of 20 µL containing 10 µL of 2 × Brilliant III SYBR Green QPCR master mix, 25 pmol of each primer, and deionized water. The PCR conditions were as follows: initial denaturation at 95 °C for 10 min and 35 cycles of denaturation, annealing, and elongation at 95 °C for 30 s, 64 °C for 30 s, and 72 °C for 30 s, respectively, followed by a cooling step at 40 °C for 30 s. The SYBR Green signals of the amplified products were acquired using the AriaMx real‐time PCR (Agilent Technologies).^17^, ^18^ To measure the cellular DNA background, the *β‐actin* gene was amplified (400 bp) using real‐time PCR. When the cellular DNA contaminated the cfDNA pools, the Ct value from real‐time PCR was lower than that of the noncontaminated samples. The PCR conditions were as follows: initial denaturation at 95 °C for 10 min and 40 cycles of denaturation, annealing, and elongation at 95 °C for 10 s, at 55 °C for 20 s, and at 72 °C for 20 s, respectively, followed by a cooling step at 40 °C for 30 s. The SYBR Green signals of the amplified products were acquired using the AriaMx real‐time PCR (Agilent Technologies). The primer sets of genes used in this study are described in Table S1 of the Supporting Information.

*Clinical Samples and WES*: To develop the simple and low‐cost cfNA‐sampling platform for clinical use, primary tissues and blood plasma were collected from 14 CRC patients, blood samples from 10 healthy controls, frozen plasma samples from 11 CRC patients (with OncoPanel results from the primary tissues), and blood plasma from five CRC patients (no primary tissue testing result) from the BRC of the Asan Medical Center (Seoul, Korea) after approval from the Institutional Review Board (IRB_ S2017‐0042‐0001). The samples were obtained by a colorectal surgical team and were randomly selected according to cancer stage. The characteristics of all patients and mutation information based on WES results of the 14 tissue samples are described in Table 1. Samples of cfDNA obtained from the patients' plasma were analyzed for hot‐spot mutations using SCODA (Theragen, Suwon, Korea).^25^

*Application of the Simple and Low‐Cost ctDNA Analysis Method*: 11 blood plasma samples obtained from CRC patients, which contained *BRAF* and *KRAS* mutations, were tested as detected using the OncoPanel. All ctDNA samples were isolated from the plasma samples using the DTBP platform that was combined with Sanger sequencing for simple and low‐cost ctDNA analyses. For the Sanger sequencing, all ctDNA samples were amplified using the sequencing primer of the *BRAF* exon 15 (annealing temperature, 58 °C) and *KRAS* exon 2 (annealing temperature, 55 °C). The samples were directly sequenced using BigDye Terminal chemistry with the forward sequencing primer of the detectable *BRAF* and *KRAS* mutations. The DNA sequencing reaction mixtures were electrophoresed using the ABI's 3730XL DNA analyzer (Applied Biosystems, USA) at the Macrogen Sequencing Center (Macrogen Inc., Seoul, Korea). The DTBP platform was used to conventional PCR methods for simple and low‐cost ctDNA analyses. To compare the sensitivity of mutation detection between the column‐based method and the DTBP platform, isolated ctDNA from blood plasma samples from five CRC patients in which mutations had not been identified were amplified with *KRAS* mutation specific primer sets, and mutations were identified using PCR and Sanger sequencing. For conventional PCR, 5 µL of DNA was amplified in a total volume of 25 µL, containing 10 × PCR buffer (Qiagen), 2.5 × 10^−3^ m MgCl_2_, 0.25 × 10^−3^ m deoxynucleotide triphosphate, 25 pmol of each primer, and 1 unit of Taq DNA polymerase (Qiagen). The PCR program for the *KRAS* mutation was performed as follows: initial denaturation at 95 °C for 15 min and 45 cycles of denaturation, annealing, and elongation at 95 °C for 30 s, 58 °C (for G12D) and 60 °C (for G13D) for 30 s, and 72 °C for 30 s, respectively, followed by final elongation at 72 °C for 7 min. Gel electrophoresis was used to separate the PCR products on a 2% agarose gel containing ethidium bromide. The gel was visualized using a Gel Doc system (Clinx Science Instruments).

*Biooptical Sensor*: The detailed preparation and operation of the biooptical sensor was the described previously.^24^

## Conflict of Interest

The authors declare no conflict of interest.

## Supporting information

Supplementary
