# Supplementary material for: Simple and Low‐Cost Sampling of Cell‐Free Nucleic Acids from Blood Plasma for Rapid and Sensitive Detection of Circulating Tumor DNA
Source: Adv Sci (Weinh). 2018 Jul 30;5(10):1800614. doi: 10.1002/advs.201800614 (PMC6193143; doi:10.1002/advs.201800614)
Supplement: Supplementary file 1 — Supplementary [file ADVS-5-1800614-s001.pdf]

Copyright WILEY-VCH Verlag GmbH & Co. KGaA, 69469 Weinheim, Germany, 2018.

ADVANCED SCIENCE
Open Access

Supporting Information

for *Adv. Sci.,* DOI: 10.1002/advs.201800614

Simple and Low-Cost Sampling of Cell-Free Nucleic Acids from Blood Plasma for Rapid and Sensitive Detection of Circulating Tumor DNA

Choong Eun Jin, Bonhan Koo, Tae Yoon Lee, Kyudong Han, Seok Byung Lim, In Ja Park, and Yong Shin**

Supplementary

Simple and low-cost sampling of cell-free nucleic acids from blood plasma for rapid and sensitive detection of circulating tumor DNA

Choong Eun Jin[1], Bonhan Koo[1], Tae Yoon Lee[2], Kyudong Han[3], Seok Byung Lim[4], In Ja Park[4,*], Yong Shin[1,*]

[1]Department of Convergence Medicine, Asan Medical Center, University of Ulsan College of Medicine, Biomedical Engineering Research Center, Asan Institute of Life Sciences, Asan Medical Center, 88 Olympicro-43gil, Songpa-gu, Seoul, Republic of Korea

[2]Department of Technology Education and Department of Biomedical Engineering, Chungnam National University, 99 Daehak-ro, Yuseong-gu, Daejeon 34134, Republic of Korea

[3]Department of Nanobiomedical Science & BK21 PLUS NBM Global Research Center for Regenerative Medicine, Dankook University, Cheonan 31116, Republic of Korea

[4]Department of Colon & Rectal Surgery, Asan Medical Center, University of Ulsan College of Medicine, Seoul, Republic of Korea

* Correspondence and request for materials should be addressed to Y. Shin (shinyongno1@gmail.com) and I.J. Park (ipark@amc.seoul.kr)

Supplementary Table 1- 3

Supplementary Figure 1-2

Supplementary Table 1. Primer sets used for conventional PCR and Sanger sequencing.

**Target gene**		**Sequence (5’ → 3’)**
Alu	247 bp	F: GTG GCT CAC GCC TGT AAT C
		R: CAG GCT GGA GTG CAG TGG
	115 bp	F: CCT GAG GTC AGG AGT TCG AG
		R: CCC GAG TAG CTG GGA TTA CA
β-actin		F: GCA CCA CAC CTT CTA CAA TGA
		R: TGT CAC GCA CGA TTT CCC
BRAF	Exon 15	F: TCA TAA TGC TTG CTC TGA TAG GA
		R: GGC CAA AAT TTA ATC AGT GGA
KRAS	G12D	F: TGT GGT AGT TGG AGC TGG
		R: TCA TGA AAA TGG TCA GAG AAA CC
	G13D	F: TGT GGT AGT TGG AGC TGG TGA G
		R: TCA TGA AAA TGG TCA GAG AAA CC
	Exon 2	F: TCA TTA TTT TTA TTA TAA GGC CTG CT
		R: CAA GAT TTA CCT CTA TTG TTG GAT C
18S rRNA		F: GCT TAA TTT GAC TCA ACA CGG GA
		R: AGC TAT CAA TCT GTC AAT CCT CTC
GADPH		F: CCT GGA GAA ACC TGC CAA GTA TG
		R: AGA GTG GGA GTT GCT TTG AAG TC

Supplementary Table 2. Comparison of the characteristics of cfDNA isolated using the column-based method and the DTBP platform from 14 colorectal cancer (CRC) samples and 10 healthy control (CTL) samples.

	Nr.	Concentration (ng/μℓ)		Integrity (*Alu* 247/115 ratio)		*β-actin* C_T	
		Column	DTBP	Column	DTBP	Column	DTBP
CRC (Patients)	T1	0.574	1.12	0.18	0.14	29.95	37.98
	T2	0.297	1.13	0.26	0.06	31.57	39.19
	T3	1.10	1.34	0.31	0.03	30.71	37.25
	T4	0.626	1.08	0.05	0.06	31.93	34.96
	T5	0.882	1.09	0.12	0.12	30.46	37.43
	T6	0.616	0.641	0.15	0.15	30.19	33.91
	T7	0.667	0.786	0.15	0.12	29.7	34.98
	T8	0.337	1.01	0.08	0.03	33.52	37.9
	T9	0.763	1.09	0.04	0.03	31.76	38.61
	T10	0.298	1.46	0.36	0.12	32.11	35.18
	T11	0.271	0.98	0.26	0.08	31.65	38.84
	T12	0.217	0.686	0.08	0.06	33.96	38.3
	T13	0.237	1.39	0.06	0.04	32.77	34.93
	T14	0.248	1.13	0.35	0.1	30.47	37.28
CTL (Healthy)	C1	0.307	0.529	0.38	0.29	30.29	39.11
	C2	0.278	1.06	0.28	0.34	30.89	38.97
	C3	0.427	0.403	0.58	0.57	28.18	35.84
	C4	0.361	0.406	0.77	0.38	28.15	36.83
	C5	0.259	0.191	0.65	0.28	30.35	36.81
	C6	0.509	0.523	0.56	0.38	25.96	35.09
	C7	0.596	0.578	0.98	0.29	27.49	35.36
	C8	0.472	0.592	0.85	0.13	28.07	36.85
	C9	0.404	0.937	0.51	0.23	28.58	37.94
	C10	0.265	0.681	0.33	0.3	30.55	37.09

Supplementary Table 3. Results of simple and low-cost ctDNA analyses of samples from 11 colorectal cancer patients using the DTBP platform and Sanger sequencing.

Nr.	Tissue OncoPanel		ctDNA Sanger sequencing		ctDNA Biosensor	
	BRAF	*KRAS*	*BRAF*	*KRAS*	*BRAF*	*KRAS*
**S1**	WT	**G12D**	WT	**G12D**	WT	**G12D**
**S2**	WT	**G12D**	WT	**G12D**	WT	**G12D**
S3	WT	WT	WT	WT	WT	WT
**S4**	WT	**G12D**	WT	WT	WT	**G12D**
**S5**	WT	**G12V**	WT	**G12V**	WT	**G12V**
S6	WT	WT	WT	WT	WT	WT
**S7**	**V600E**	WT	**V600E**	WT	**V600E**	WT
S8	WT	WT	WT	WT	WT	WT
**S9**	WT	**G12V**	WT	WT	WT	WT
**S10**	WT	**G13D**	WT	**G13D**	WT	**G13D**
S11	WT	WT	WT	WT	WT	WT

Bold text indicates detected mutations.

WT: wild type

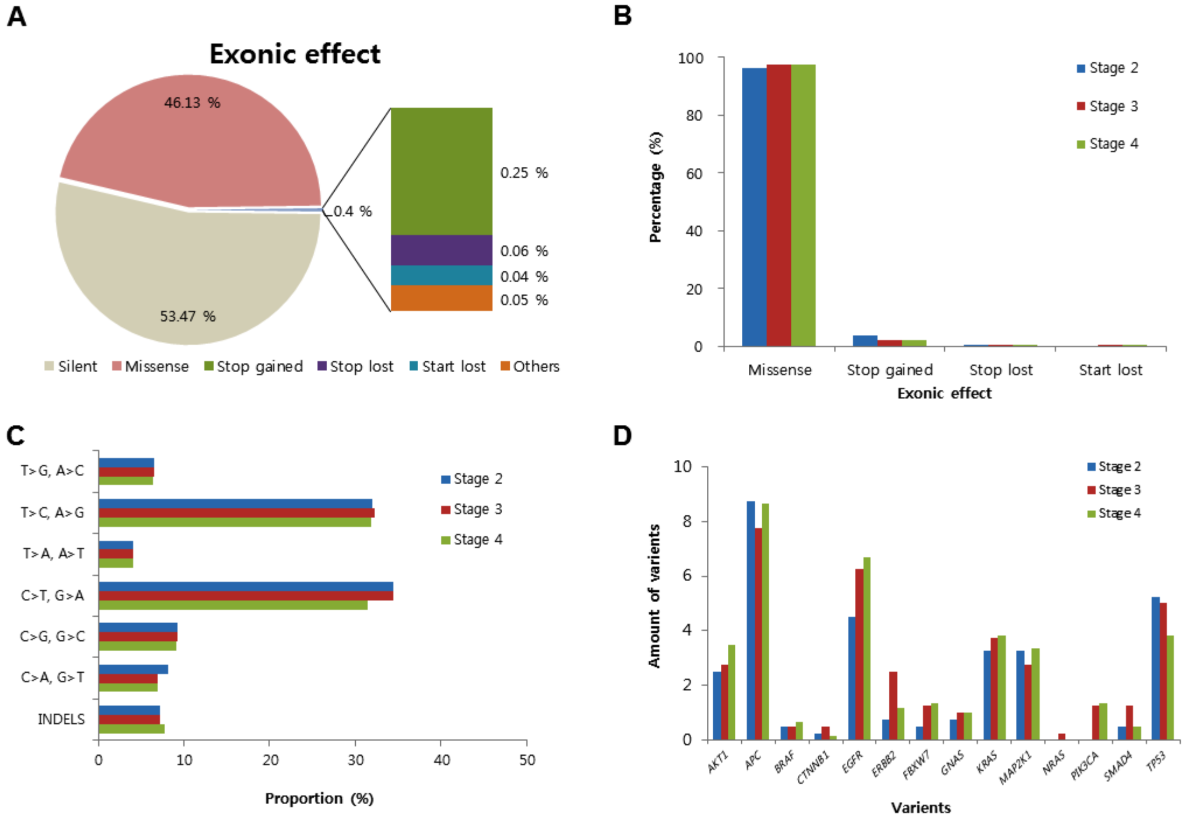

Supplementary Figure 1. Genetic characteristics of 14 primary tissue samples from colorectal cancer patients determined using WES. A) Number of exonic effects from 14 tissues. B) Exonic effect dependence on the cancer stage of colorectal cancer (CRC) patients. C) Dependence of the number of nucleotide substitution classes on the cancer stage. D) Dependence on mutation frequencies on the cancer stage in CRC samples.

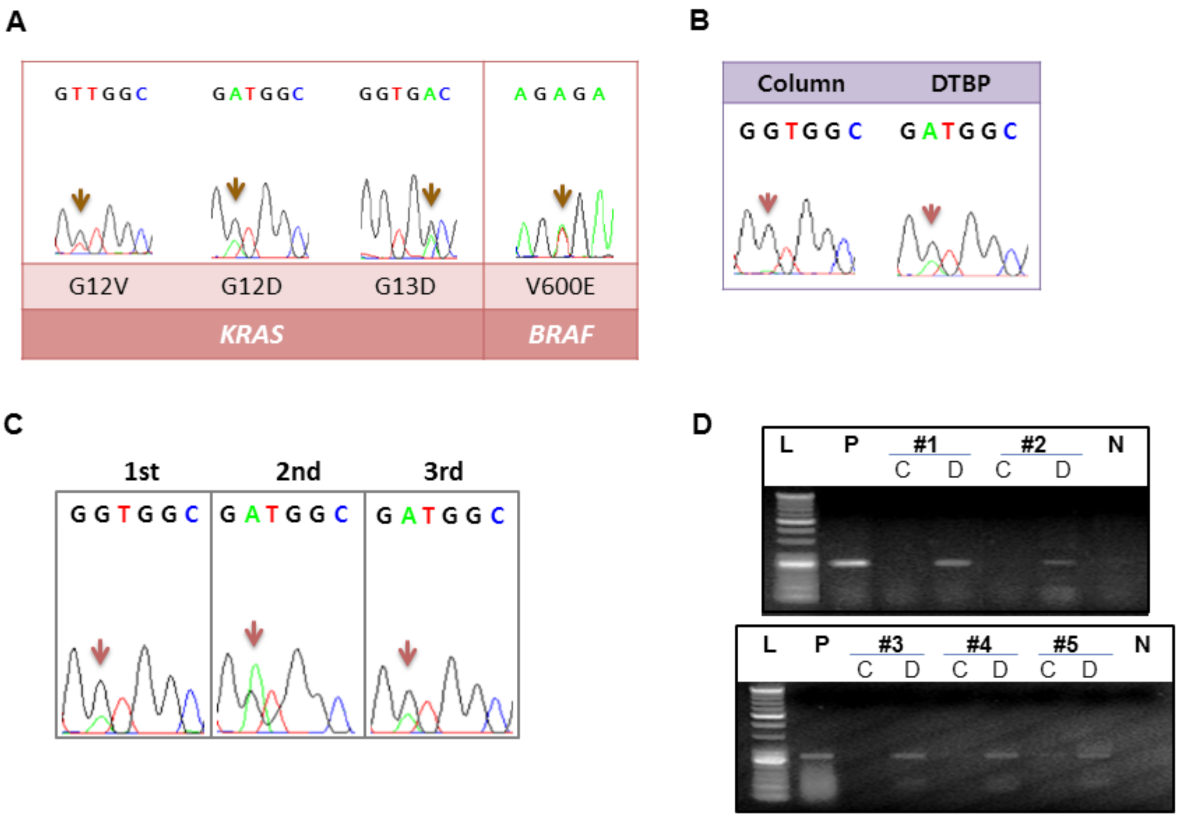

Supplementary Figure 2. Simple and low-cost ctDNA analysis for clinical diagnosis. A) ctDNA was isolated from 11 plasma samples of colorectal cancer patients using the DTBP platform, and the Sanger sequencing was then performed for ctDNA analysis. The mutation sequence identified using the DTBP platform. B) Sequencing results of ctDNA for the detection of the *KRAS* mutation using the column-based method and DTBP platform. C) Reproducibility of the DTBP platform with a different sample tube of the same patient. D) Gel electrophoretic analysis of the PCR products with *KRAS* G12D (left) and *KRAS* G13D (right) mutations extracted from five CRC patients using the column-based method and the DTBP platforms (L: 500bp DNA ladder, P: positive control, #1-2: plasma samples for the G12D mutation, #3–5: plasma samples for the G13D mutation, C: column-based method, D: DTBP platform, and N: negative control).
